# The *i-Rheo* Framework: A Unified Approach to Broadband Rheology and Microrheology of Soft Matter from Molecular Simulations to Biomechanics

**DOI:** 10.3390/mi17101197

**Published:** 2026-10-16

**Authors:** Manlio Tassieri

**Affiliations:** Division of Biomedical Engineering, James Watt School of Engineering, Advanced Research Centre, University of Glasgow, Glasgow G11 6EW, UK; manlio.tassieri@glasgow.ac.uk

**Keywords:** rheology, broadband rheology, linear viscoelasticity, microrheology, soft matter, i-Rheo, time–frequency transformation, optical tweezers, indentation, molecular dynamics

## Abstract

Linear viscoelasticity provides a time-invariant mechanical fingerprint of materials and underpins predictive descriptions of their response to external deformation. The corresponding material functions reflect the hierarchy of inter- and intra-molecular interactions governing structure and dynamics across multiple scales, from atomistic motions to macromolecular and mesoscopic rearrangements. Access to linear viscoelastic properties over the widest possible frequency range therefore offers unique insight into the mechanisms controlling the behaviour of soft matter and complex fluids. However, obtaining reliable broadband viscoelastic spectra remains experimentally and analytically challenging owing to instrumental limitations, finite measurement windows, discrete data sampling, and the shortcomings of conventional transformation procedures. This Review/Perspective examines the evolution of the *i-Rheo* Framework, a model-free methodology for extracting broadband viscoelastic properties directly from finite experimental or simulated datasets without imposing predefined constitutive models. Originally developed to transform creep-compliance measurements into the complex shear modulus, the framework has subsequently been extended to bulk rheology, passive and active microrheology, optical tweezers, atomic force microscopy, macro-indentation, biomechanics, rheo-optical measurements, and molecular simulations. The recent development of *i-Rheo-Tempo* further establishes a reversible connection between time- and frequency-domain representations, providing a unified analytical framework for multiscale viscoelastic characterisation across soft-matter physics.

## 1. Introduction

Rheology underpins virtually every process involving the deformation, flow, or processing of soft matter. From polymer manufacturing and food production to pharmaceuticals, cosmetics, biological tissues, and living cells, knowledge of a material’s rheological properties is fundamental to understanding its behaviour, predicting its performance, and optimising its processing conditions [1,2].

Among these properties, the linear viscoelastic (LVE) response occupies a particularly important position. When measured within the linear regime, it provides a mechanical fingerprint of the material and, importantly, reflects the molecular and supramolecular dynamics responsible for its macroscopic behaviour. Different relaxation processes occur over different characteristic timescales and therefore manifest themselves at different frequencies. Consequently, the information contained in an LVE measurement becomes increasingly valuable as the experimentally accessible frequency window is widened.

Small-amplitude oscillatory shear has long represented the gold standard for determining the frequency-dependent storage and loss moduli, $G^{\prime }\left(\omega \right)$ and $G^{\prime \prime }\left(\omega \right)$. However, conventional frequency sweeps are intrinsically sequential: each frequency must be probed individually. The accessible bandwidth is therefore constrained at low frequencies by increasingly long measurement times and at high frequencies by instrument inertia and transducer dynamics. Similar limitations arise in microrheology, where the accessible window is ultimately determined by acquisition time, temporal resolution, detector bandwidth, and signal-to-noise ratio [3].

Broadband linear viscoelasticity does not, however, necessarily require oscillatory excitation. In a linear system, a transient perturbation contains information over a continuum of frequencies. Creep and stress-relaxation experiments can therefore encode a broad viscoelastic spectrum within a single measurement. The fundamental challenge is to recover this frequency-domain information directly from finite, discrete, and imperfect experimental time-series without imposing a preconceived constitutive model or introducing artefacts through numerical transformations.

An early solution to this problem demonstrated that experimentally measured creep compliance could be converted directly into $G^{\prime }\left(\omega \right)$ and $G^{\prime \prime }\left(\omega \right)$, without fitting the data to a predefined rheological model or resorting to Laplace/inverse-Laplace transformations [4]. This concept was subsequently generalised in *i-Rheo*, where the actual time-dependent stress and strain histories generated during a step-strain experiment are transformed directly to recover the LVE spectrum over the widest experimentally accessible frequency range [5].

What began as a solution to a specific time–frequency transformation problem has progressively developed into what I refer to here as the *i-Rheo* Framework: a common approach for extracting broadband linear viscoelastic information directly from experimentally accessible observables. Over the years, this principle has been extended from bulk rheometry to micro- and nano-rheological measurements, including particle-tracking and optical-tweezers microrheology, atomic force microscopy (AFM) based measurements, indentation and rheo-optical approaches, spanning synthetic soft materials and biological systems.

In this Review/Perspective, I revisit the foundations and evolution of the *i-Rheo* Framework, focusing not on a chronological catalogue of individual methods, but on the common principles that connect them across experimental platforms, material classes, length scales, and time/frequency scales. I finally discuss how the recent development of *i-Rheo-Tempo* completes the transformation from time to frequency and back to time and consider the opportunities and limitations of this framework for the future of broadband rheological characterisation.

## 2. The *i-Rheo* Principle: From Raw Experimental Data to Material Functions

### 2.1. The Origins: From Creep Compliance to Dynamic Moduli

The mathematical origin of the *i-Rheo* Framework can be traced to the problem of converting an experimentally measured creep compliance, J(t), directly into the corresponding frequency-dependent viscoelastic moduli [4]. In linear viscoelasticity, the relaxation modulus G(t) and creep compliance J(t) satisfy

(1) $$\int_{0}^{t}G\left(\tau \right)J\left(t-\tau \right)\;d\tau =t,$$

which, upon Fourier transformation (denoted hereafter by a hat, ^^^), gives

(2) $$G^{*}\left(\omega \right)=i\omega \hat{G}\left(\omega \right)=\frac{1}{i\omega \hat{J}\left(\omega \right)}.$$

Thus, knowledge of J(t) is, in principle, sufficient to determine the complete frequency-dependent LVE response. The practical difficulty is that experimental creep data are available only over a finite time window, are generally non-uniformly sampled, and that J(t) itself may not converge at long times.

The key development introduced by Evans et al. [4] was to reformulate the transformation in terms of the second derivative of the measured function. For a generic causal function f(t),

(3) $$\hat{f}\left(\omega \right)=\frac{1}{{\left(i\omega \right)}^{2}}F\left\{\ddot{f}\left(t\right)\right\},$$

provided that causality and the appropriate boundary contributions are explicitly retained. For a finite set of experimental data $\left(t_{k},f_{k}\right)$, with k=1,…,N, the resulting analytical expression can be written as

(4) $$\begin{array}{cc} {\left(i\omega \right)}^{2}\hat{f}\left(\omega \right)= & i\omega f\left(0\right)+\left(1-e^{-i\omega t_{1}}\right)\frac{f_{1}-f\left(0\right)}{t_{1}}+{\dot{f}}_{\infty }e^{-i\omega t_{N}}\\ \; & +\sum_{k=2}^{N}\left(\frac{f_{k}-f_{k-1}}{t_{k}-t_{k-1}}\right)\left(e^{-i\omega t_{k-1}}-e^{-i\omega t_{k}}\right), \end{array}$$

where f(0) is the value extrapolated to t=0 from above, and ${\dot{f}}_{\infty }$ is the gradient extrapolated to infinite time.

For creep compliance, this formulation is particularly advantageous because J(t) approaches a linear function at long times for a fluid, whereas $\ddot{J}\left(t\right)$ vanishes. The transformation is therefore shifted from the original experimental function to one whose Fourier transform is well behaved.

Equation (4) is the central mathematical result of the Evans solution. It transforms the experimentally sampled function directly, retaining the actual sampling times and the relevant boundary contributions, without requiring the data to be fitted to a predefined constitutive model. Although its form may initially appear non-intuitive, each term follows directly from the geometry of a piecewise-linear representation of the experimental data, as shown in the following subsection.

### 2.2. The Pivotal Role of the Second Derivative

The origin of Equation (4) becomes particularly transparent when the experimental data are viewed geometrically. Consider a generic function f(x) known only at the discrete points ${\left\{x_{k},f_{k}\right\}}_{k=0}^{N}$. The simplest continuous representation that passes exactly through every measured point is piecewise linear:

(5) $$f\left(x\right)=a_{k}x+b_{k},\;a_{k}=\frac{f_{k+1}-f_{k}}{x_{k+1}-x_{k}},\;x_{k}\leq x<x_{k+1}.$$

**Figure 1:**
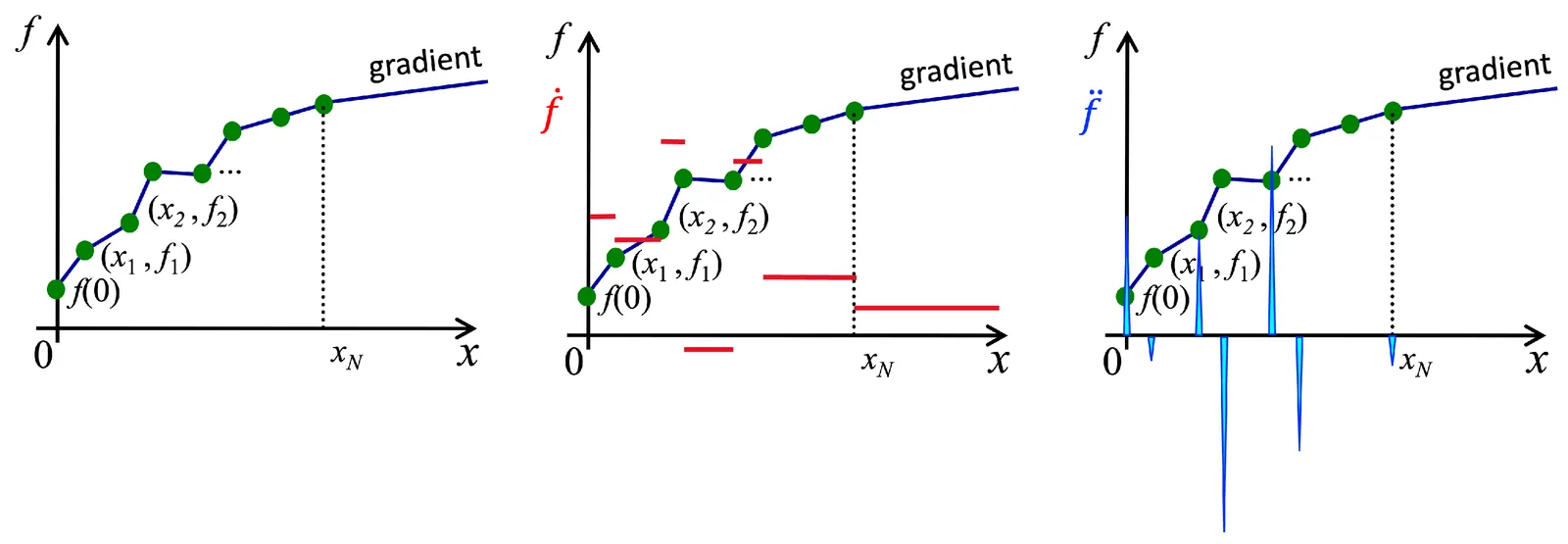
Schematic representation of a generic discretely sampled function f(x) and its distributional derivatives. The sampled data are connected by piecewise-linear segments, with f(0) finite and a finite asymptotic gradient as x→+∞. The first derivative, $\dot{f}\left(x\right)$, is piecewise constant, while the second derivative, $\ddot{f}\left(x\right)$, vanishes between sampling points and reduces to a series of Dirac delta functions at the nodes, with amplitudes determined by the changes in local gradient.

As illustrated in Figure 1, the first derivative of the piecewise-linear representation is piecewise constant. Its second derivative therefore vanishes between adjacent data points and is non-zero only where the local gradient changes. In the distributional sense,

(6) $$\ddot{f}\left(x\right)=\sum_{k}\Delta a_{k}\;\delta \left(x-x_{k}\right),\;\Delta a_{k}=a_{k}-a_{k-1},$$

with the appropriate additional contributions arising from the behaviour at the boundaries.

This is the pivotal mathematical simplification behind Equation (4). Instead of Fourier transforming the original experimental function, one transforms a finite series of Dirac delta functions located at the experimental sampling points. Their sifting property converts the Fourier integral into an analytical sum,

(7) $$F\left\{\ddot{f}\left(t\right)\right\}=\sum_{k}\Delta a_{k}e^{-i\omega t_{k}},$$

to which the initial and long-time boundary contributions are added. Substituting the slopes of the individual piecewise-linear segments then leads directly to Equation (4).

This construction has a close mathematical analogy with Green’s-function methods. The second derivative acts as a linear operator that maps the piecewise-linear representation onto a discrete set of Dirac sources located at changes in gradient. Conversely, the original piecewise-linear function can be regarded as a superposition of shifted fundamental solutions of the second-derivative operator, supplemented by the appropriate boundary terms. The *i-Rheo* approach exploits the complementary direction of this correspondence: by reducing the measured function to its distributional second derivative, an integral transformation is converted into a finite superposition of elementary contributions that can be evaluated analytically.

The importance of this result is that the transformation is constructed from the experimental data themselves. The actual sampling times are retained and need not be equally spaced; no numerical quadrature is required; and no predefined relaxation spectrum or constitutive model is imposed.

### 2.3. From Discrete Data to Reliable Transformations: Virtual Oversampling and DIP

Although the Evans solution can operate directly on irregularly spaced data, its accuracy ultimately depends on how faithfully the piecewise-linear representation captures the underlying continuous response. By construction, the second derivative of this representation vanishes between consecutive sampling points. Any curvature of the actual response occurring between those points is therefore not represented and cannot contribute to the transformation. This limitation becomes increasingly important as the experimental sampling becomes sparse relative to the variations of the measured function.

To improve the numerical representation of such datasets, a *virtual-oversampling* procedure was introduced within the *i-Rheo* methodology [6,7]. The measured data are first interpolated by a suitable continuous function and then resampled at a much higher density before applying the analytical transformation. Virtual oversampling does not generate new experimental information; rather, it provides a denser representation of the response inferred from the original measurements.

The interpolation itself is important because the *i-Rheo* transformations are integral in nature and their analytical reformulation exploits derivatives of the represented function. The interpolant must therefore provide a sufficiently regular representation without introducing artificial oscillations, overshoots or curvature that could propagate into the transformed response. The influence of the interpolation scheme was investigated systematically in *i-RheoFT*, together with sampling density and experimental noise [7].

To quantify the information contained in the original dataset, *i-RheoFT* introduced the *density of initial points* (DIP) [7],

(8) $$\mathrm{DIP}=\frac{\log_{10}\left(N_{e}\right)}{\log_{10}\left(t_{N}/t_{1}\right)},$$

where $N_{e}$ is the number of experimentally acquired points within the time window $t_{1}\leq t\leq t_{N}$. DIP therefore provides a dimensionless measure relating the number of experimentally acquired points to the logarithmic span of the observation window and is distinct from the virtual-oversampling factor, β. Increasing β can improve the numerical representation of an adequately sampled response, but it cannot compensate for insufficient initial sampling [7].

The results of *i-RheoFT* also provide practical guidance on the sampling requirements. For the two representative functions investigated there, DIP values were varied between 0.25 and 1. For the corresponding frequency-domain moduli, all three interpolation schemes considered returned mean relative absolute errors below 1% for DIP >0.4 in the test case examined, corresponding to approximately 40 initial data points across the four-decade observation window. This value should not, however, be regarded as a universal threshold: the required DIP depends on the shape and complexity of the sampled function, the interpolation method and the signal-to-noise ratio. Insufficient initial sampling can generate interpolation overshoots or distortions which subsequently appear as spurious features in the transformed spectrum, particularly towards the limits of the experimentally accessible frequency window. Virtual oversampling cannot recover information that was absent from the original measurements.

Experimental noise represents a related limitation. The analytical nature of the transformation does not suppress noise present in the input data, and errors introduced during interpolation can propagate into the recovered frequency-domain response. The systematic analysis performed with *i-RheoFT* showed that noise-induced errors in the interpolation of the shortest-time behaviour primarily contaminate the high-frequency end of the transformed spectrum. At relatively low signal-to-noise ratios, shape-preserving interpolation schemes such as PCHIP and Makima were found to be more robust than cubic splines, whereas the differences become negligible for sufficiently well-resolved, high-signal-to-noise data. Consequently, neither virtual oversampling nor the analytical transformation should be regarded as a substitute for adequate experimental sampling and signal quality. The same consideration applies to the inverse frequency-to-time transformation implemented in *i-Rheo-Tempo* [8]: features reconstructed in G(t) can only be as reliable as the finite, noisy frequency-domain data from which they originate.

These considerations extend naturally beyond the original time-to-frequency problem. Because both forward and inverse transformations involve integral relations, their fidelity is ultimately governed by the information density, interpolation and finite boundaries of the measured function. This symmetry is made explicit by *i-Rheo-Tempo*, which extends the second-derivative strategy to the inverse frequency-to-time transformation [8]. Thus, analytical exactness does not remove the fundamental requirement for sufficiently informative experimental data.

It is important to clarify that, throughout the *i-Rheo* Framework, the term *model-free* refers specifically to the rheological transformation: no predefined viscoelastic constitutive equation or relaxation spectrum is imposed to recover the material function. It does not imply that the underlying experiment is free from physical, geometrical or instrumental assumptions. The relation between the measured observables and the corresponding mechanical response remains technique-dependent and may rely, for example, on probe–medium coupling and continuum assumptions in microrheology, contact mechanics in indentation or an appropriate force–deformation relation in direct mechanical measurements. Application of the framework to a new system therefore requires verification that the experiment operates within the linear-response regime, that the relevant geometry and boundary conditions are adequately described, and that instrumental contributions do not dominate the measured response. Only under these conditions can the recovered response be interpreted as an intrinsic material property rather than as an effective response of the complete experimental configuration.

In practice, these requirements can be assessed through straightforward experimental checks. Linearity can be examined by repeating measurements at different small deformation amplitudes and verifying the independence of the recovered response from the imposed amplitude. Geometrical and boundary effects can be investigated by varying probe size, indentation depth, sample thickness or measurement location, as appropriate to the technique. Instrumental contributions can be assessed through calibration, reference measurements and checks for inertia, drift and finite detector bandwidth.

The importance of identifying the linear-response regime is also illustrated by the Pipkin diagram presented in the recent British Society of Rheology review [9] (Figure 1a therein), which places *i-Rheo* [5] and optimally windowed chirp rheometry [10] in the broader context of linear and nonlinear rheometric protocols.

## 3. From a Mathematical Principle to the *i-Rheo* Framework

### 3.1. From Creep Compliance to Bulk Rheology

The first experimental demonstration of the Evans solution was deliberately performed on a demanding benchmark system: a near-monodisperse linear polyisoprene melt ($M_{w}/M_{n}=1.03$), whose relaxation dynamics could be independently characterised by conventional rheometry. The complex modulus obtained directly from the measured creep compliance, without smoothing or fitting to a rheological model, agreed with conventional oscillatory measurements over approximately five decades in frequency [4]. This established the original transformation

(9) $$J\left(t\right)⟶G^{*}\left(\omega \right),$$

and demonstrated that a single transient experiment can encode essentially the same LVE information that would otherwise require a sequence of oscillatory measurements.

A major conceptual extension came with the introduction of *i-Rheo* in 2016 [5]. Rather than requiring the experimental response to be reduced first to a particular material function such as J(t), *i-Rheo* operates directly on the measured stress σ(t) and strain γ(t) histories generated during a real step experiment. Within the linear viscoelastic regime, the complex modulus follows from

(10) $$G^{*}\left(\omega \right)=\frac{\hat{\sigma }\left(\omega \right)}{\hat{\gamma }\left(\omega \right)},$$

where both Fourier transforms are evaluated directly from the experimentally sampled functions using the analytical procedure described in Section 2. Crucially, the experimentally realised deformation does not need to be replaced by an ideal mathematical step: the actual input γ(t) and output σ(t) are transformed together. Thus, the original idea was generalised from

(11) $$J\left(t\right)⟶G^{*}\left(\omega \right)\;⟹\;\left\{\sigma \left(t\right),\gamma \left(t\right)\right\}⟶G^{*}\left(\omega \right).$$

**Figure 2:**
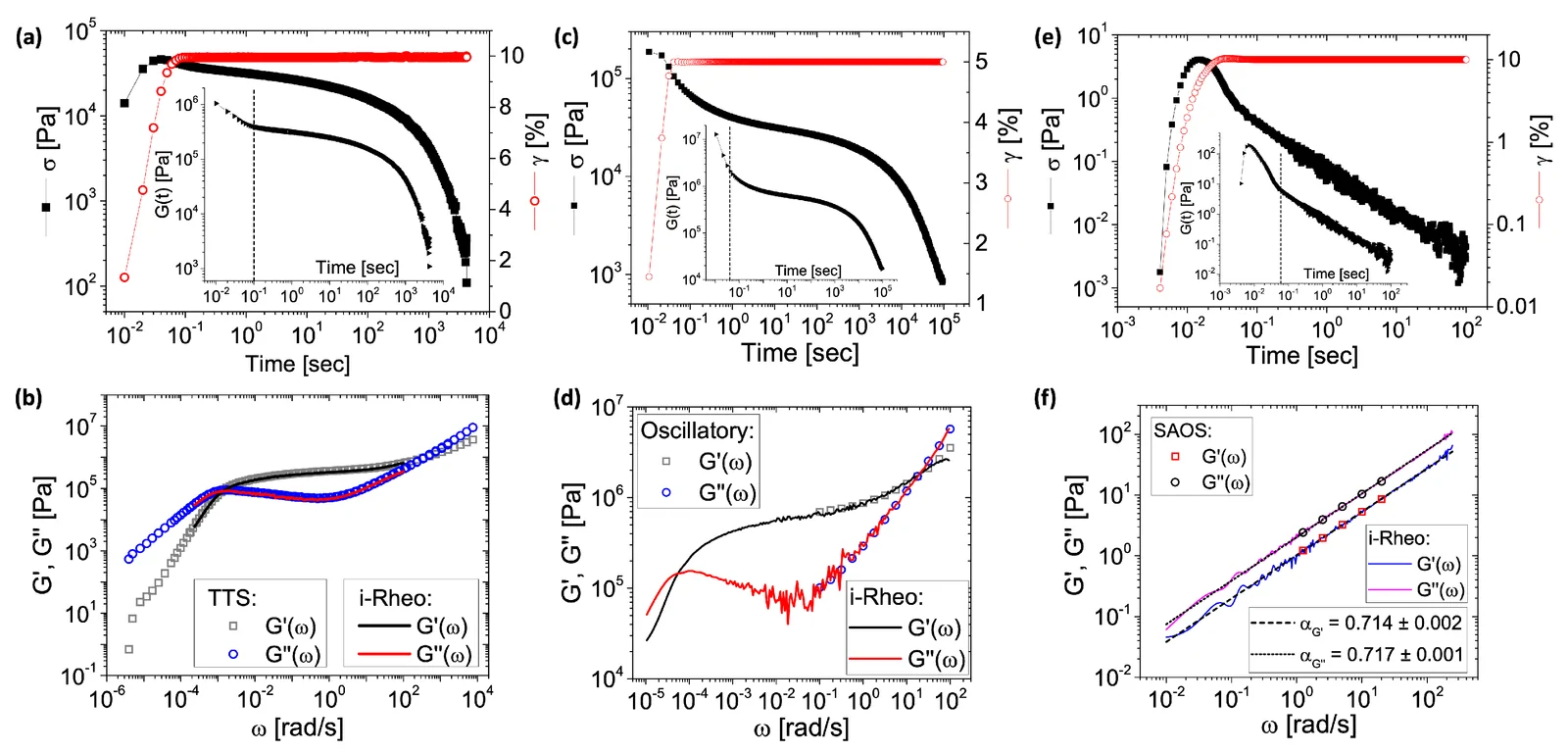
Generality of *i-Rheo* across distinct classes of viscoelastic materials. Selected examples from the broader range of materials investigated: (**a**,**b**) a monodisperse linear polyisoprene melt, (**c**,**d**) an industrial styrene–butadiene rubber (SBR), and (**e**,**f**) an aqueous gelatin solution at the gel point. (**Top**) experimentally measured stress and strain histories during the imposed step deformation. The insets show the corresponding relaxation modulus, conventionally evaluated as G(t)=σ(t)/γ; the vertical dashed lines indicate the short-time cut-off before which the imposed strain has not yet reached its constant value and the data would therefore be discarded in a conventional stress-relaxation analysis. (**Bottom**) storage and loss moduli obtained directly from the transient measurements using *i-Rheo* (lines) compared with conventional oscillatory measurements or time–temperature superposition (symbols). Adapted from Ref. [5].

The generality of this approach was deliberately tested on materials exhibiting markedly different rheological behaviour, including a monodisperse linear polyisoprene melt, a bimodal polyisoprene blend, an industrial styrene–butadiene rubber (SBR), an aqueous gelatin solution at the gel point, and a highly concentrated colloidal suspension [5]. Representative examples are shown in Figure 2. These systems span a wide spectrum of material classes and relaxation dynamics, from well-defined entangled polymer melts, whose behaviour is accurately described by established polymer theories and validated through time–temperature superposition, to industrial elastomers, concentrated colloids, and ultra-soft materials undergoing sol–gel transitions. Despite these profound differences in structure and dynamics, the same model-free transformation was successfully applied without modifying the underlying analytical formalism or introducing material-specific constitutive assumptions.

In this sense, the significance of *i-Rheo* extended beyond the possibility of replacing a conventional frequency sweep with a step experiment. It demonstrated that broadband rheological information could be extracted directly from the actual transient input and output of a linear mechanical experiment, largely independently of the microscopic origin or magnitude of the material response. This shift—from transforming a specific rheological function, J(t), to transforming experimentally accessible observables—provided the conceptual step from the Evans solution towards the broader *i-Rheo* Framework.

Beyond these original demonstrations, the methodology was subsequently adopted independently in markedly different areas of soft-matter rheology. For instance, Silva et al. applied the Evans/*i-Rheo* approach to vitreous humour, converting creep-compliance measurements of its gel phase into $G^{\prime }\left(\omega \right)$ and $G^{\prime \prime }\left(\omega \right)$, with results consistent with independent small-amplitude oscillatory shear measurements [11]. In a very different context, Xie et al. employed *i-Rheo* to transform stress-relaxation measurements of super-soft bottlebrush polymers, extending their experimentally accessible viscoelastic spectrum towards substantially lower frequencies [12]. These independent applications, spanning biological gels and advanced polymeric materials, illustrate the broader applicability of the same model-free transformation beyond the systems on which it was originally demonstrated.

### 3.2. Microrheology: From Probe Motion to Broadband Viscoelasticity

Microrheology extends rheological measurements to microscopic length scales by using the motion of micron-sized probes to interrogate the mechanical response of their local environment. Depending on whether this motion arises from thermal fluctuations or is induced by an externally applied force, microrheological techniques are conventionally classified as *passive* or *active*, respectively [3]. Regardless of the approach, the primary experimental observable is the time-dependent trajectory of the probe, whereas the quantity ultimately sought is the frequency-dependent complex shear modulus, $G^{*}\left(\omega \right)$. Microrheology therefore inherently requires a transformation from a finite, discretely sampled time-domain experimental signal to the frequency domain. This provides a natural connection with the *i-Rheo* Framework, whose analytical treatment of discrete time-domain data offers a model-free route to $G^{*}\left(\omega \right)$.

In passive microrheology, the probe undergoes Brownian motion driven by thermal fluctuations of the surrounding medium. When particle inertia can be neglected, the fluctuation–dissipation theorem establishes a direct correspondence between the mean-square displacement (MSD) of a freely diffusing spherical probe and the macroscopic creep compliance of the surrounding material [13],

(12) $$\langle \Delta r^{2}\left(\tau \right)\rangle =\frac{k_{B}T}{\pi a}\;J\left(\tau \right),$$

where τ is the lag time, $k_{B}$ is the Boltzmann constant, *T* is the absolute temperature, and *a* is the probe radius. Thus, the thermal energy $k_{B}T$ provides the driving energy, while the resulting stochastic motion of the probe encodes the creep compliance of its local environment. The connection with the *i-Rheo* Framework is therefore immediate:

$$\mathrm{probe}\;\mathrm{trajectory}⟶\mathrm{MSD}\left(\tau \right)\propto J\left(\tau \right)⟶\mathrm{analytical}\;\mathrm{transformation}⟶G^{*}\left(\omega \right).$$

Optical tweezers provide a particularly powerful implementation of this principle by confining the Brownian probe within an approximately harmonic potential. The corresponding generalized Langevin equation becomes

(13) $$ma\left(t\right)=f_{R}\left(t\right)-\int_{0}^{t}\zeta \left(t-\tau \right)v\left(\tau \right)\;d\tau -\kappa r\left(t\right),$$

where *m* is the probe mass, r(t) is its displacement from the trap centre, v(t)=dr(t)/dt and a(t)=dv(t)/dt are its velocity and acceleration, respectively, $f_{R}\left(t\right)$ is the stochastic thermal force, ζ(t) is the memory kernel describing the time-dependent friction exerted by the surrounding viscoelastic medium, and κ is the optical-trap stiffness. The convolution integral accounts for the history-dependent drag experienced by the probe.

**Figure 3:**
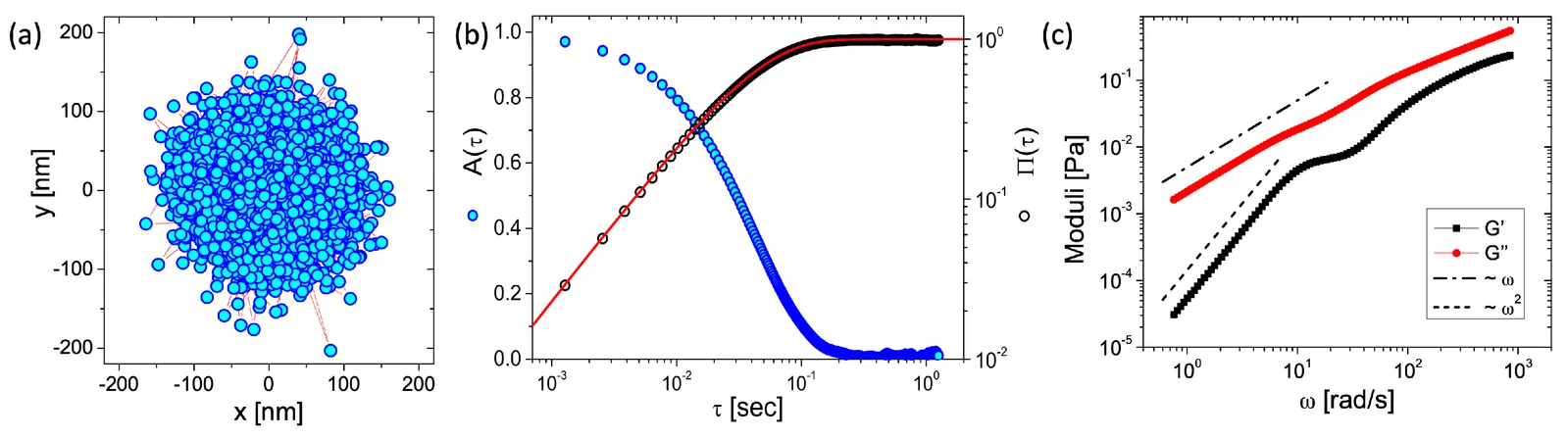
Passive optical-tweezers microrheology. (**a**) Typical thermally driven trajectory of a microsphere confined within an optical trap. (**b**) The measured trajectory is analysed in the time domain to determine the normalised mean-square displacement (NMSD), Π(t), and the normalised position autocorrelation function (NPAF), A(t). (**c**) Analytical transformation of these observables yields the frequency-dependent storage and loss moduli, $G^{\prime }\left(\omega \right)$ and $G^{\prime \prime }\left(\omega \right)$, respectively.

Neglecting particle inertia, the solution of Equation (13) relates the complex shear modulus $G^{*}\left(\omega \right)$ directly to either the normalised mean-square displacement (NMSD), $\Pi \left(t\right)=\langle \Delta r^{2}\left(t\right)\rangle /\left[2\langle r^{2}\rangle \right]$, or the normalised position autocorrelation function (NPAF), $A\left(t\right)=\langle r\left(0\right)r\left(t\right)\rangle /\langle r^{2}\rangle$, where $\langle r^{2}\rangle$ is the equilibrium positional variance of the trapped probe, and the angular brackets denote an ensemble, or equivalently under ergodic conditions, a time average [6]:

(14) $$\frac{6\pi a}{\kappa }G^{*}\left(\omega \right)=\left[\frac{1}{i\omega \hat{\Pi }\left(\omega \right)}-1\right]={\left[\frac{1}{i\omega \hat{A}\left(\omega \right)}-1\right]}^{-1},$$

where *a* is the probe radius, ω is the angular frequency, *i* is the imaginary unit. The two representations are equivalent and can therefore be analysed using the same analytical transformation strategy introduced in Section 2. The complete procedure, from the measured Brownian trajectory to the time–domain observables and the corresponding frequency–dependent viscoelastic moduli, is illustrated in Figure 3.

The analytical methodology has subsequently been adopted independently to investigate the mechanics of biomolecular condensates. Alshareedah et al. used passive optical-tweezers microrheology to show that protein–RNA condensates behave as Maxwell fluids whose viscoelastic response can be tuned through polypeptide and RNA sequence and composition [14]. The methodology was later applied to protein condensates to relate sequence-specific molecular interactions to their viscoelasticity and ageing dynamics [15]. More recently, Zhou et al. employed passive optical-tweezers microrheology to characterise the viscoelastic properties of chromatin condensates, linking their mechanical response to chromatin structure and intermolecular interaction networks [16]. These studies illustrate the application of the same analytical framework to increasingly complex biological materials.

In active microrheology, by contrast, an external force deliberately drives the probe through the material, producing a displacement substantially larger than the underlying thermal fluctuations [17]. For optical tweezers this can be achieved by moving the centre of the optical trap, $r_{c}\left(t\right)$, so that the generalized Langevin equation becomes

(15) $$ma\left(t\right)=f_{R}\left(t\right)-\int_{0}^{t}\zeta \left(t-\tau \right)v\left(\tau \right)\;d\tau +\kappa \left[r_{c}\left(t\right)-r\left(t\right)\right].$$

The resulting complex modulus is obtained from the relationship between the imposed motion of the trap and the measured response of the probe [18],

(16) $$\frac{6\pi a}{\kappa }G^{*}\left(\omega \right)=\frac{{\hat{r}}_{c}\left(\omega \right)}{\langle \hat{r}\left(\omega \right)\rangle }-1,$$

where $r_{c}\left(t\right)$ is the prescribed trajectory of the trap centre, r(t) is the resulting probe trajectory and the angular brackets denote an ensemble average over repeated measurements. Since the imposed trap motion is assumed to be reproducible, ${\hat{r}}_{c}\left(\omega \right)$ is identical for each realisation and does not require ensemble averaging, whereas the probe response contains a stochastic contribution from thermal fluctuations and therefore enters as $\langle \hat{r}\left(\omega \right)\rangle$. Particle inertia has been neglected in Equation (16). Importantly, this expression is not restricted to sinusoidal forcing: the imposed trap trajectory may have an arbitrary temporal form within the linear-response regime.

**Figure 4:**
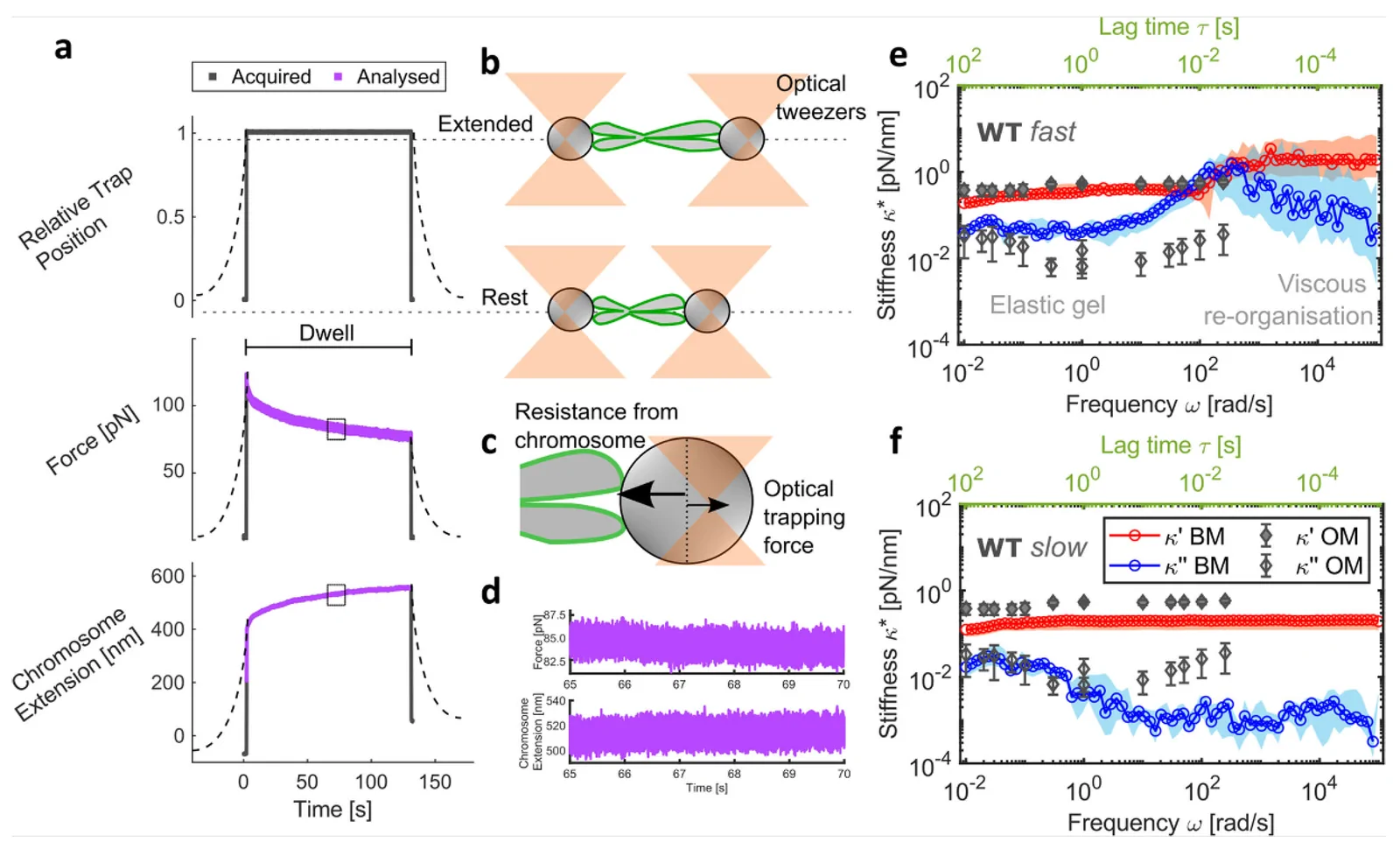
Broadband microrheology of individual mitotic chromosomes. (**a**) Stretch–hold optical-tweezers protocol showing the imposed trap displacement and the resulting force and chromosome-extension signals; the purple data acquired during the dwell period are used for broadband analysis. (**b**) Schematic of the chromosome suspended between two optically trapped bead handles in the extended and resting configurations. (**c**) Opposing chromosome resistance and optical-trapping forces acting on a bead handle. (**d**) Representative high-frequency force and chromosome-extension signals during the dwell period. (**e**,**f**) Elastic, $\kappa^{\prime }\left(\omega \right)$, and viscous, $\kappa^{\prime \prime }\left(\omega \right)$, components of the complex chromosome stiffness obtained from broadband microrheology (BM) at fast (100μm $s^{-1}$) and slow (0.2μm $s^{-1}$) force-loading rates, respectively, overlaid with oscillatory microrheology (OM) data from Meijering et al. [20]. The fast stretch reveals a high-frequency regime of viscous reorganisation followed, at longer timescales, by predominantly elastic gel-like behaviour, whereas the slower loading predominantly probes the gel-like response. Adapted from Ref. [19].

A further extension of the framework was recently demonstrated by Mendonca et al., who adapted the same analytical principle to direct mechanical measurements of individual mitotic chromosomes using optical tweezers [19]. As illustrated in Figure 4, a chromosome suspended between two optically trapped beads was subjected to a rapid stretch–hold deformation, while the resulting force, F(t), and chromosome extension, λ(t), were measured simultaneously. Their analytical Fourier transforms were then used to determine the chromosome complex stiffness,

(17) $$\kappa^{*}\left(\omega \right)=\frac{\hat{F}\left(\omega \right)}{\hat{\lambda }\left(\omega \right)}=\kappa^{\prime }\left(\omega \right)+i\kappa^{\prime \prime }\left(\omega \right).$$

Implemented through *i-Rheo C-Stretch*, this single transient measurement provided the mechanical response of individual chromosomes over approximately seven decades of frequency, revealing a transition from high-frequency viscous reorganisation to predominantly elastic, gel-like behaviour at longer timescales [19]. The approach therefore extends the *i-Rheo* concept beyond conventional probe-based microrheology: rather than inferring the rheology of the medium surrounding a probe, the optical tweezers directly deform the microscopic biological structure itself, while the same time-to-frequency analytical framework is retained.

### 3.3. From Nano- to Macro-Indentation: Broadband Rheology Across Length Scales

Indentation methods, and atomic force microscopy in particular, are widely used to characterise soft materials, biological matter and individual cells. Conventionally, a single force–indentation curve is often interpreted through contact-mechanics models, such as the Hertz model, to obtain an elastic parameter such as the Young’s modulus. While this provides useful information on material stiffness, for intrinsically viscoelastic materials, it leaves uncharacterised the viscous, energy-dissipating component of the mechanical response and its dependence on timescale [21,22]. This missing information can be particularly important in biological systems: materials having essentially the same Young’s modulus can exhibit markedly different viscous responses, which can alter cell adhesion and mechanotransduction and ultimately influence cell fate [23]. More generally, the extraction of a Young’s modulus from a single indentation curve relies on fitting the indentation ramp over a selected deformation range and can therefore be sensitive to the determination of the contact point, the chosen fitting interval, indentation rate and the assumptions underlying the contact model [24].

An alternative is to treat indentation as a linear-viscoelastic experiment. Early work by Tripathy and Berger formulated stress-relaxation indentation by combining Hertzian contact mechanics with the Boltzmann superposition principle, although the resulting relaxation response was interpreted by fitting prescribed viscoelastic models [25]. For a spherical indenter, the force response to an arbitrary indentation history can instead be written as

(18) $$P\left(t\right)=\int_{-\infty }^{t}G\left(t-\tau \right)\frac{d\Lambda [\delta (\tau )]}{d\tau }\;d\tau ,$$

where P(t) is the measured indentation force, G(t) is the shear relaxation modulus, δ(t) is the indentation depth and, for a rigid spherical indenter of radius *R*, $\Lambda \left[\delta \left(t\right)\right]=8\sqrt{R}\;\delta^{3/2}\left(t\right)/\left[3\left(1-\nu \right)\right]$, with ν the Poisson ratio. Fourier transformation therefore gives directly

(19) $$G^{*}\left(\omega \right)=\frac{\hat{P}\left(\omega \right)}{\hat{\Lambda }\left(\omega \right)},$$

providing the same input–output structure encountered throughout the *i-Rheo* Framework.

**Figure 5:**
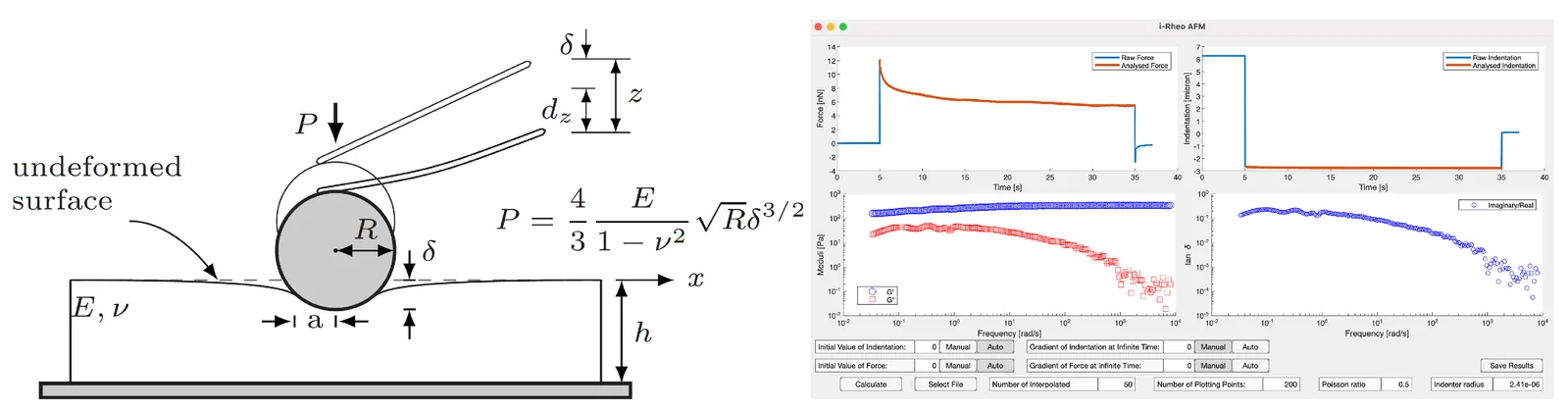
Model-free broadband rheology from AFM step-indentation. (**Left**) schematic of spherical AFM indentation, where the measured force P(t) and indentation depth δ(t) are related through the contact geometry and the material viscoelastic response. (**Right**) *i-Rheo AFM* analysis of the measured force and indentation histories following a step-indentation, yielding directly the frequency-dependent storage and loss moduli, $G^{\prime }\left(\omega \right)$ and $G^{\prime \prime }\left(\omega \right)$, and the loss tangent, tanδ, without assuming a predefined viscoelastic constitutive model. A freely available GUI implementing the analysis can be downloaded from the University of Glasgow Research Data Repository: https://researchdata.gla.ac.uk/1604/ (accessed on 11 October 2026).

This principle formed the basis of *i-Rheo AFM* (Figure 5), where a rapid step-indentation followed by stress relaxation was used to recover $G^{\prime }\left(\omega \right)$ and $G^{\prime \prime }\left(\omega \right)$ of soft materials and living cells directly from the measured force and indentation histories, without assuming a viscoelastic constitutive model [21]. The approach provided a continuous viscoelastic spectrum over approximately five decades of frequency and was subsequently developed as model-free Rheo-AFM to probe the viscoelasticity of individual dsDNA soft colloids [22]. Importantly, the step-indentation protocol also avoids the need for continuous oscillatory indentation: once the target indentation is reached, the deformation is held constant and the subsequent force relaxation contains the time-dependent material response.

**Figure 6:**
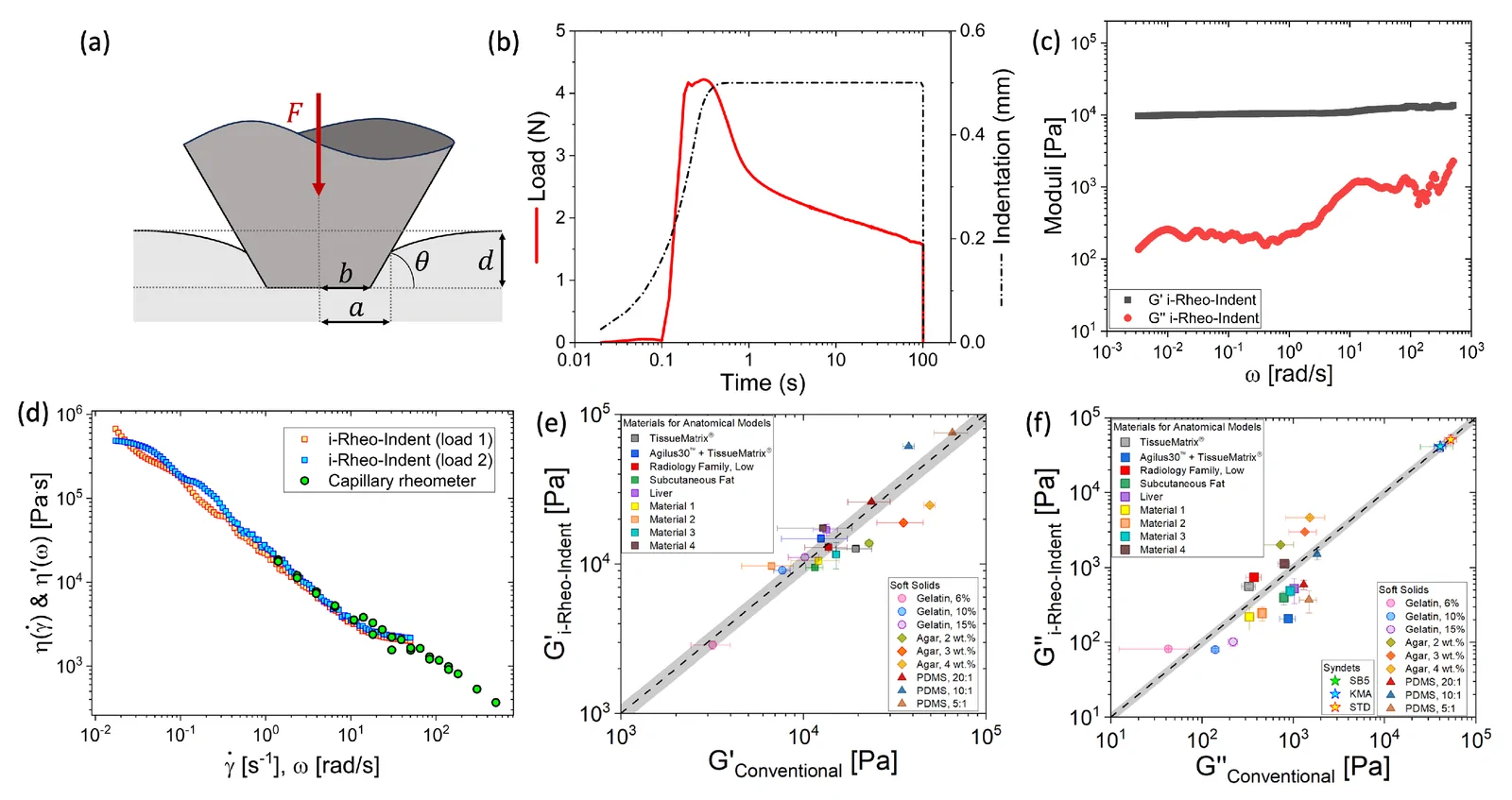
*i-Rheo-Indent*: model-free broadband rheology by macroscale indentation. (**a**) Schematic of the indentation geometry using a rigid truncated-conical indenter. (**b**) Representative step-indentation experiment showing the imposed indentation depth and the resulting time-dependent load relaxation. (**c**) Storage and loss moduli, $G^{\prime }\left(\omega \right)$ and $G^{\prime \prime }\left(\omega \right)$, obtained by analytically transforming the measured indentation and load histories without assuming a predefined viscoelastic constitutive model. (**d**) Comparison of the real part of the complex viscosity, $\eta^{\prime }\left(\omega \right)$, obtained from two independent *i-Rheo-Indent* measurements with the steady-shear viscosity, $\eta (\dot{\gamma })$, measured by capillary rheometry for a representative syndet formulation. (**e**,**f**) Overall validation of *i-Rheo-Indent* across the investigated material classes: comparison of the storage modulus, $G^{\prime }$, and loss modulus, $G^{\prime \prime }$, respectively, with values obtained by conventional oscillatory rheometry for hydrogels, polydimethylsiloxane (PDMS) elastomers, anatomical modelling materials and syndet formulations. The dashed line indicates one-to-one agreement and the grey band a deviation of ±15%. Adapted from Ref. [26].

More recently, the *i-Rheo* methodology was extended from AFM nanoindentation to macroscale testing through *i-Rheo-Indent* [26]. As shown in Figure 6, a rapid step indentation is applied using a rigid truncated-conical indenter (Figure 6a,b), and the measured load-relaxation response and indentation depth are treated as conjugate time-domain observables. Their direct analytical transformation yields the storage and loss moduli, $G^{\prime }\left(\omega \right)$ and $G^{\prime \prime }\left(\omega \right)$, without oscillatory excitation or predefined viscoelastic constitutive models (Figure 6c). The methodology was validated across a broad range of soft materials, including hydrogels, PDMS elastomers, anatomical modelling materials, and syndet formulations, showing excellent agreement with conventional oscillatory rheometry (Figure 6e,f). Beyond its analytical simplicity, *i-Rheo-Indent* offers important practical advantages for the rheological characterisation of soft solids. Unlike capillary rheometry, it requires only a simple pill-shaped specimen rather than the relatively large sample volumes needed to fill a capillary barrel, while a single experiment provides broadband information that would otherwise require multiple measurements to span a comparable frequency or shear-rate range. For example, the complex viscosity obtained from independent *i-Rheo-Indent* measurements closely reproduces steady-shear capillary-rheometry data for representative syndet formulations (Figure 6d). Combined with simplified sample preparation and cleaning procedures, this single-measurement approach reduced the overall experimental time by more than a factor of thirty compared with conventional rheological characterisation [26]. Furthermore, unlike rotational rheometry, indentation-based measurements are largely insensitive to sample–tool contact issues and wall slip, which can compromise the characterisation of soft solids [27,28]. Thus, although the experimental implementation spans length scales from nanometre-scale AFM probing to millimetre-scale indentation, the underlying analytical framework remains unchanged.

### 3.4. From Experiments to Simulations: i-Rheo GT

The same mathematical problem encountered in experimental rheology also arises in molecular simulations. In equilibrium molecular dynamics, the linear viscoelastic response is commonly obtained as the time-dependent shear relaxation modulus, G(t), evaluated at a discrete set of time points over a finite simulation window. Although G(t) contains the material’s linear viscoelastic response, a more complete picture of its rheological behaviour is provided by the frequency dependence of the storage and loss moduli. These are related to the relaxation modulus through

(20) $$G^{*}\left(\omega \right)=i\omega \hat{G}\left(\omega \right).$$

Consequently, extracting broadband rheological information from molecular simulations poses essentially the same problem encountered experimentally: the Fourier transformation of a function known only through a finite and discretely sampled time-domain data set. This observation provided the basis for extending the *i-Rheo* Framework from experimental measurements to molecular simulations through *i-Rheo GT* [29].

Conventionally, simulated G(t) data have often been transformed into the frequency domain by first fitting them with a generalised Maxwell model, whose analytical Fourier transform can then be evaluated straightforwardly. Although widely used, this procedure introduces several potential sources of uncertainty: the fitting can be non-unique, its outcome depends on the number of Maxwell modes selected by the operator, and the extrapolation beyond the simulation window relies on the strong assumption that the unresolved long-time relaxation remains exponential. Moreover, the fitting procedure can fail to preserve information contained in the short-time behaviour of G(t), where fast molecular relaxation processes are encoded. *i-Rheo GT* removes this intermediate modelling step by applying the analytical transformation introduced in Section 2 directly to the discrete G(t) data, recovering $G^{\prime }\left(\omega \right)$ and $G^{\prime \prime }\left(\omega \right)$ without assuming a predefined relaxation spectrum [29].

**Figure 7:**
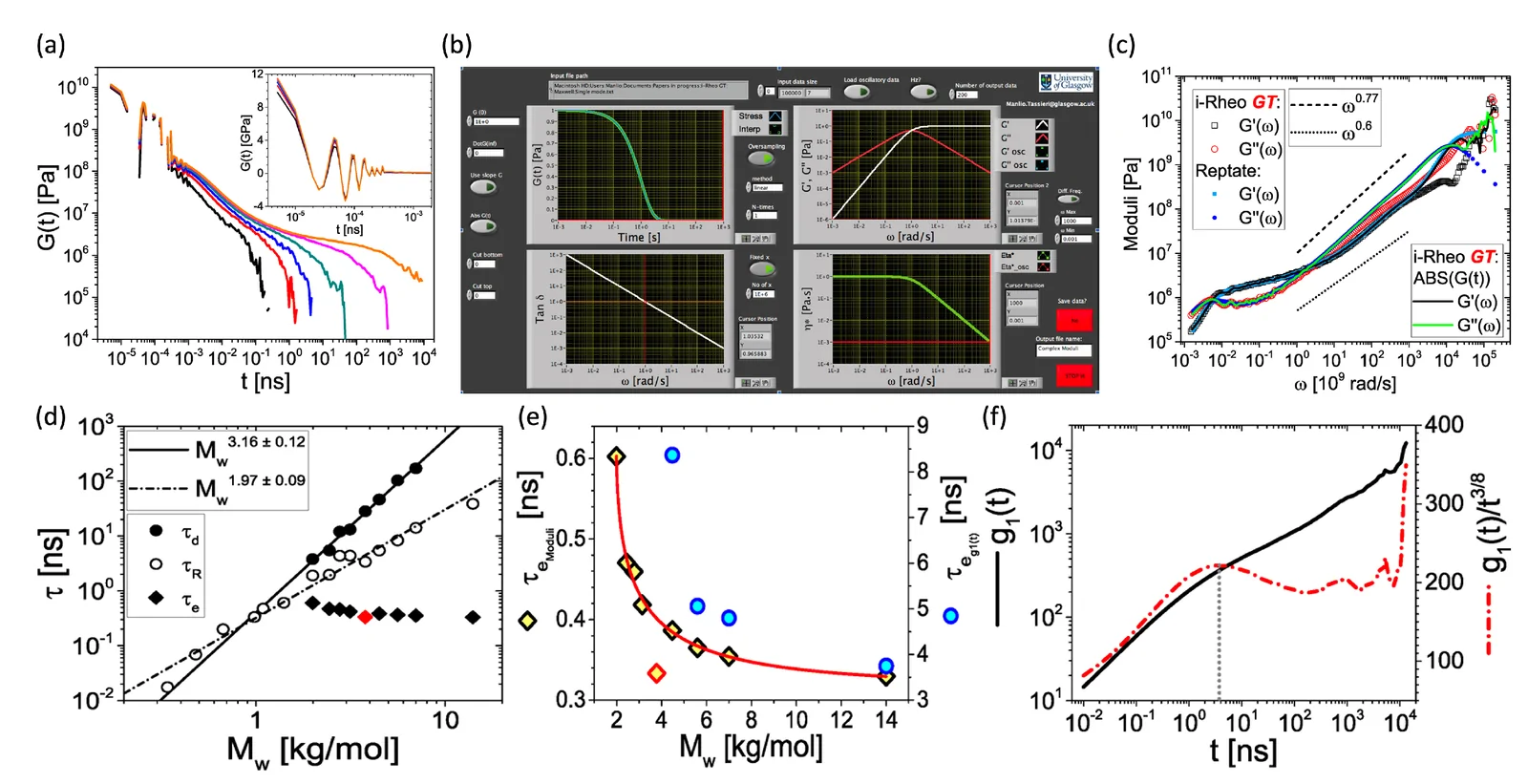
*i-Rheo GT*: from molecular-dynamics simulations to broadband rheology. (**a**) Shear relaxation modulus, G(t), obtained from atomistic molecular-dynamics simulations of linear polyethylene melts of different molecular weights; the inset highlights the short-time oscillatory response arising from molecular vibrations and inertial effects. (**b**) Original *i-Rheo GT* interface for the direct transformation of simulated G(t) into frequency-dependent viscoelastic moduli. (**c**) Comparison of $G^{\prime }\left(\omega \right)$ and $G^{\prime \prime }\left(\omega \right)$ obtained from the same atomistic simulation using *i-Rheo GT* and a generalised-Maxwell fitting approach (Reptate), revealing increasing differences in the high-frequency response; transformation of |G(t)| is also shown for comparison. (**d**) Molecular-weight dependence of the characteristic entanglement, Rouse and disengagement times, $\tau_{e}$, $\tau_{R}$ and $\tau_{d}$, extracted from the resulting viscoelastic spectra. (**e**) Comparison of the entanglement time obtained rheologically from the high-frequency crossover of $G^{\prime }\left(\omega \right)$ and $G^{\prime \prime }\left(\omega \right)$ with that independently estimated from the middle-monomer mean-square displacement. (**f**) Representative middle-monomer mean-square displacement, $g_{1}\left(t\right)$, and $g_{1}\left(t\right)/t^{3/8}$ used to identify the entanglement time. Adapted from Ref. [29].

The advantages of this model-free transformation become particularly evident when analysing atomistic molecular-dynamics simulations, where the short-time response contains information on molecular processes that can be difficult to represent through a discrete relaxation spectrum. This was demonstrated for linear polyethylene melts spanning different molecular weights [29]. As shown in Figure 7, the simulated G(t) extends from fast, strongly oscillatory molecular dynamics to the much slower relaxation processes associated with polymer-chain motion. For an entangled melt with $M_{w}=5.6$ kg ${\mathrm{mol}}^{-1}$, transformation of the same G(t) using *i-Rheo GT* and a generalised-Maxwell fitting approach produced essentially equivalent moduli from approximately $10^{-3}$ to 1 rad ${\mathrm{ns}}^{-1}$, but increasingly different responses at higher frequencies. The discrepancy emerged around the inverse entanglement time, $1/\tau_{e}$, within the rubber-to-glass transition region where Rouse and glassy relaxation processes coexist. At still higher frequencies, the Maxwell-based transformation could not reproduce the complex response associated with the short-time oscillations of G(t), arising from damped bonded interactions and inertial effects in the atomistic model.

Importantly, retaining this high-frequency information enabled the resulting viscoelastic spectra to be used to identify characteristic polymer relaxation times across molecular weights, including the entanglement, Rouse and disengagement times, $\tau_{e}$, $\tau_{R}$ and $\tau_{d}$, respectively (Figure 7d). The entanglement times obtained from the high-frequency crossover of $G^{\prime }\left(\omega \right)$ and $G^{\prime \prime }\left(\omega \right)$ could also be compared with independent estimates derived from the middle-monomer mean-square displacement (Figure 7e,f). Thus, *i-Rheo GT* not only extended the transformation of simulated G(t) into the glassy regime and beyond but also enabled features of the broadband viscoelastic spectrum to be directly connected to characteristic molecular dynamics.

More generally, however, a relaxation feature or characteristic timescale identified in a broadband mechanical spectrum does not by itself uniquely identify the molecular mechanism responsible for it. Such assignments require additional physical interpretation and, wherever possible, comparison with complementary structural or dynamical observables, as illustrated here by the independent determination of the entanglement time from monomer mean-square displacements. The same consideration applies to quantities such as activation energies inferred from the temperature dependence of relaxation times: these represent an additional level of physical interpretation beyond the model-free rheological transformation itself.

The methodology has subsequently been adopted beyond its original implementation, both in rheological software and in independent molecular-simulation studies. *i-Rheo GT* has been incorporated into RepTate, providing a direct route from G(t) to $G^{\prime }\left(\omega \right)$ and $G^{\prime \prime }\left(\omega \right)$, and was independently implemented in the commercial multiscale molecular- simulation platform J-OCTA for the transformation of relaxation moduli obtained from molecular-dynamics simulations [30,31]. Importantly, the methodology has also been used independently in molecular simulation studies. Xia, Kalow and Olvera de la Cruz employed *i-Rheo GT* to transform the stress relaxation modulus obtained from hybrid molecular-dynamics/Monte Carlo simulations of unentangled vitrimers into the corresponding frequency-dependent viscoelastic moduli [32]. This enabled direct comparison of the simulated linear rheology with predictions of the sticky Rouse model, linking microscopic molecular dynamics and bond-exchange processes to the macroscopic viscoelastic response. These independent implementations and applications illustrate how *i-Rheo GT* has evolved from an analytical methodology into a practical computational tool for connecting molecular simulations with frequency-domain rheology.

### 3.5. From Frequency Back to Time: i-Rheo-Tempo

All the developments discussed so far share the same direction of transformation: experimentally measured or simulated time-domain observables are converted into frequency-dependent viscoelastic functions. The complementary problem—recovering the time-domain response directly from experimentally measured frequency-domain data—is equally fundamental, but considerably challenging in practice because rheological spectra are necessarily finite, discretely sampled and affected by experimental noise. This inverse problem was recently addressed through *i-Rheo-Tempo* [8], which extends the same derivative-based philosophy of the i-Rheo Framework to the reconstruction of the shear relaxation modulus, G(t), from dynamic rheological measurements. Starting from the complex viscosity, $\eta^{*}\left(\omega \right)=G^{*}\left(\omega \right)/\left(i\omega \right)$, successive integrations by parts transfer the inverse Fourier transformation from the spectral functions themselves to their second derivatives. When the experimentally sampled spectrum is represented piecewise linearly, these derivatives reduce to discrete changes in local slope, allowing G(t) to be reconstructed through finite analytical sums without numerical quadrature, parametric fitting or a predefined relaxation spectrum.

**Figure 8:**
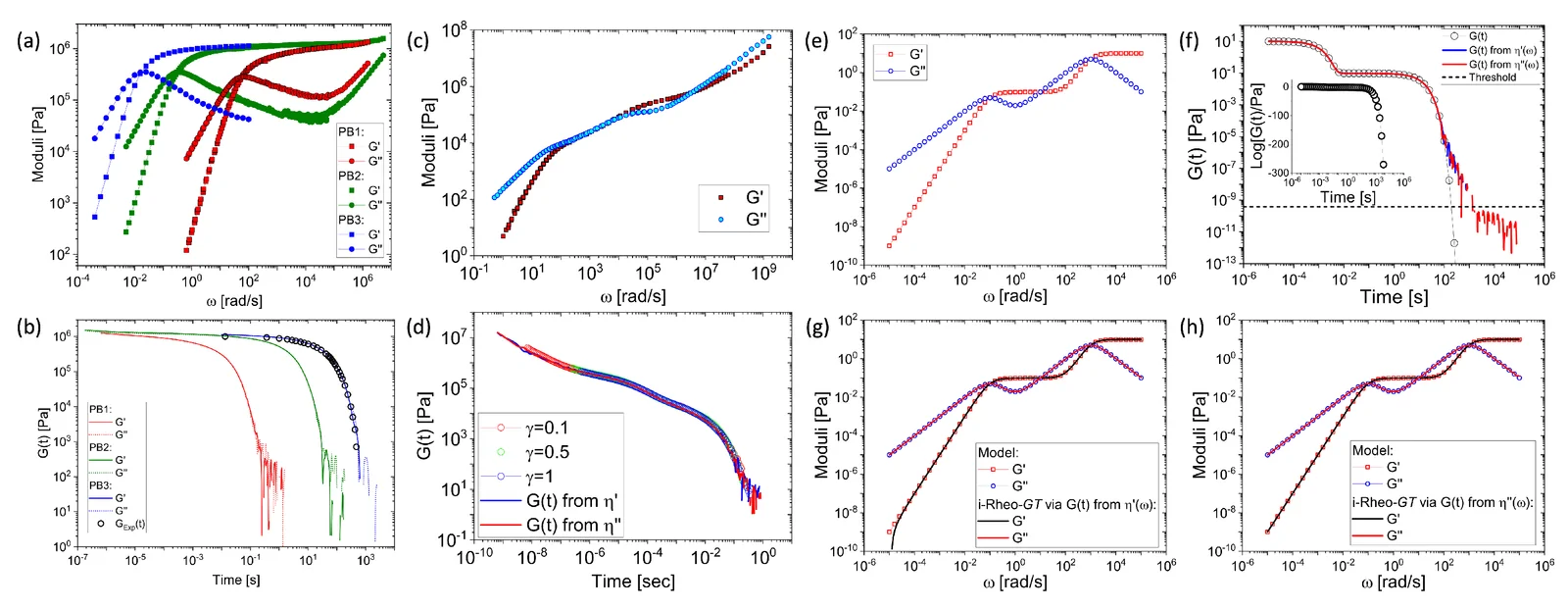
*i-Rheo-Tempo*: experimental validation and closure of the time–frequency loop. (**a**,**b**) Application to three monodisperse linear polybutadiene melts (PB1–PB3): (**a**) dynamic moduli, $G^{\prime }\left(\omega \right)$ and $G^{\prime \prime }\left(\omega \right)$, for samples of different molecular weights; (**b**) relaxation moduli, G(t), reconstructed using *i-Rheo-Tempo*, together with the experimental estimate, $G_{\exp}\left(t\right)=\sigma \left(t\right)/\gamma \left(t\right)$, available for PB3. (**c**,**d**) Application to the model comb polymer lc3-PBd1k at ϕ=0.5: (**c**) dynamic moduli obtained via time–temperature superposition; (**d**) relaxation modulus reconstructed independently from $\eta^{\prime }\left(\omega \right)$ and $\eta^{\prime \prime }\left(\omega \right)$ and compared with experimental stress-relaxation measurements performed at γ=0.1, 0.5, and 1, all within the linear viscoelastic regime. (**e**–**h**) Closed-loop validation using a Burgers model: (**e**) input dynamic moduli; (**f**) relaxation modulus reconstructed independently from $\eta^{\prime }\left(\omega \right)$ and $\eta^{\prime \prime }\left(\omega \right)$ and compared with the analytical solution, with the dashed line indicating the threshold for the onset of the unreliable long-time regime; (**g**,**h**) dynamic moduli recovered by applying *i-Rheo GT* to the relaxation moduli reconstructed from $\eta^{\prime }\left(\omega \right)$ and $\eta^{\prime \prime }\left(\omega \right)$, respectively. Recovery of the original dynamic moduli demonstrates the closed-loop consistency of the frequency-to-time and time-to-frequency transformations. Adapted from Ref. [8].

The methodology was validated across a broad range of experimental systems, including an industrial styrene–butadiene rubber, monodisperse linear polyisoprene and polybutadiene melts, and architecturally complex comb polymers exhibiting hierarchical relaxation processes [8]. Two examples are shown in Figure 8. For three monodisperse linear polybutadiene melts of different molecular weights (Figure 8a,b) [33,34], the measured dynamic moduli were transformed into G(t), with the reconstruction for PB3 showing excellent agreement with the independently available experimental time-domain response. For the model comb polymer lc3-PBd1k at ϕ=0.5 (Figure 8c,d) [35], the relaxation modulus reconstructed from the time–temperature-superposition master curve agreed closely with independent stress-relaxation measurements performed at three strain amplitudes within the linear viscoelastic regime. Importantly, the reconstruction captured both the early-time branch relaxation and the slower backbone dynamics characteristic of the comb architecture.

The sensitivity of *i-Rheo-Tempo* to experimental noise and numerical resolution has also been investigated systematically [8]. Controlled tests using synthetic Burgers and power-law–Maxwell spectra, contaminated with random noise ranging from 0% to 16%, demonstrated that the method maintains good reconstruction accuracy within the experimentally supported time window. Comparisons with the Baumgaertel–Winter and ReSpect approaches showed that the relative performance depends on the underlying relaxation spectrum and noise level.

An important limitation arises at long times, where uncertainties in the lowest-frequency slopes of the complex viscosity become increasingly influential. The original study introduced a quantitative reliability threshold based on the uncertainty of the first low-frequency interval, allowing the onset of numerically unreliable reconstruction to be identified. Furthermore, interpolation and resampling tests demonstrated that the reconstruction becomes less sensitive to the choice of interpolation scheme when the original experimental spectrum is sufficiently densely sampled. Shape-preserving interpolation can mitigate artificial oscillations, particularly for sparsely sampled spectra, but cannot recover information absent from the original measurements.

These findings establish the practical robustness of the inverse transformation while highlighting the importance of adequate spectral resolution, appropriate boundary conditioning and restricting the interpretation of the reconstructed relaxation modulus to the experimentally supported time window.

More fundamentally, *i-Rheo-Tempo* completes the analytical connection between the time- and frequency-domain representations of linear viscoelasticity. Within the broader *i-Rheo* Framework, experimentally accessible time-domain observables can be transformed into the complex shear modulus, for example,

$$\left\{\sigma \left(t\right),\gamma \left(t\right)\right\}\;\overset{\;i\text{-}\mathrm{Rheo}\;}{\to }\;G^{*}\left(\omega \right),$$

while a known relaxation modulus can be transformed directly through *i-Rheo GT*,

$$G\left(t\right)\;\overset{\;i\text{-}\mathrm{Rheo}\mathrm{GT}\;}{\to }\;G^{*}\left(\omega \right).$$

*i-Rheo-Tempo* provides the complementary transformation,

$$G^{*}\left(\omega \right)\;\overset{\;i\text{-}\mathrm{Rheo}\text{-}\mathrm{Tempo}\;}{\to }\;G\left(t\right),$$

thereby closing the time–frequency loop. This reversibility was demonstrated explicitly using a Burgers model (Figure 8e–h): G(t) was first reconstructed from the frequency-domain data using *i-Rheo-Tempo* and then transformed back into $G^{\prime }\left(\omega \right)$ and $G^{\prime \prime }\left(\omega \right)$ using *i-Rheo GT*, recovering the original dynamic moduli within the supported frequency window. This closed-loop test establishes the internal consistency of the transformation under controlled conditions; it should not be interpreted as implying immunity to experimental noise, finite bandwidth or insufficient sampling, which remain properties of the input data.

The framework was further challenged using broadband diffusing-wave-spectroscopy microrheology data spanning nearly nine decades in frequency, demonstrating that experimentally accessible frequency-domain information can be converted into time-domain material responses over an exceptionally broad range of timescales [8]. Beyond establishing the robustness of the inverse transformation, these results considerably widen the scope of the *i-Rheo* Framework: once the analytical transformation is no longer tied to a particular experimental protocol, observable, or direction of transformation, it becomes possible to consider its application to a much broader range of measurement modalities.

## 4. Recent Developments and Emerging Opportunities

The developments described above suggest that the broader potential of the *i-Rheo* Framework lies not in any particular experimental technique, but in the analytical treatment of experimentally accessible observables. Once suitable time-dependent quantities can be related to a material response, the same model-free transformation strategy can potentially be exploited across substantially different measurement modalities. Recent developments are beginning to demonstrate this broader scope.

**Figure 9:**
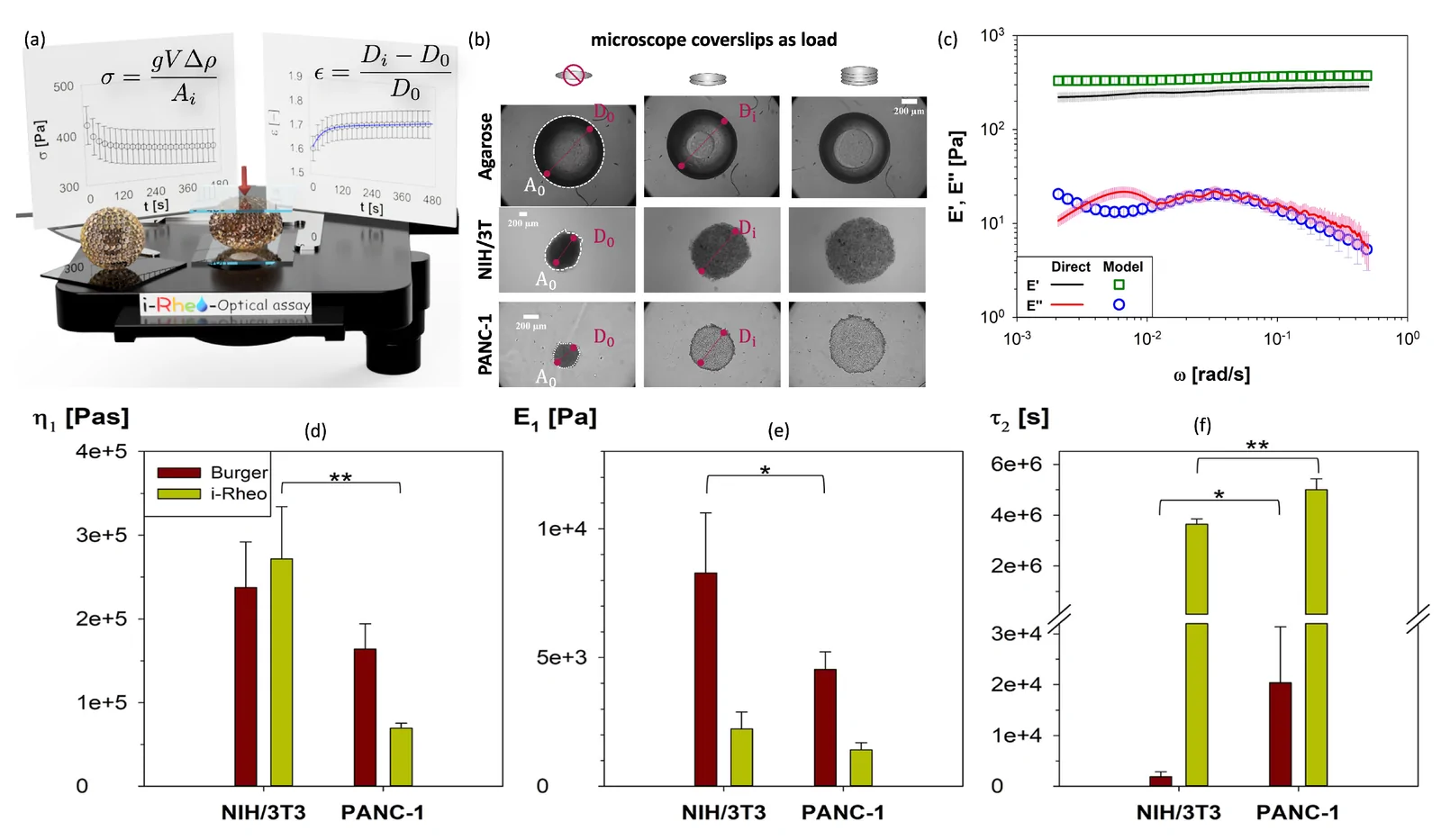
*i-Rheo-optical assay*: broadband viscoelastic characterisation of multicellular spheroids. (**a**) Concept of the rheo-optical compression assay, in which a microscope coverslip applies a constant compressive load to a multicellular spheroid while time-resolved optical microscopy tracks its morphological response, allowing the stress, σ(t), and strain, ε(t), histories to be determined. (**b**) Representative microscopy images illustrating the deformation of multicellular spheroids under compression and the image-derived geometrical quantities used to quantify their time-dependent deformation. (**c**) Representative compressional storage and loss moduli, $E^{\prime }\left(\omega \right)$ and $E^{\prime \prime }\left(\omega \right)$, obtained directly from the measured stress and strain histories using the model-free *i-Rheo* approach (lines) and compared with those obtained by fitting the same experimental data with a Burgers model (symbols). (**d**–**f**) Comparison between NIH/3T3 non-tumoral and PANC-1 pancreatic-cancer spheroids of the Burgers parameters $\eta_{1}$, $E_{1}$ and $\tau_{2}=\eta_{2}/E_{2}$, respectively, obtained either directly from Burgers-model fitting or by fitting the corresponding model to the frequency-dependent response obtained using *i-Rheo*. Here, $E_{1}$ describes the instantaneous elastic response, $\eta_{1}$ the long-time viscous flow, and $\tau_{2}$ the characteristic time associated with the retarded elastic response. Statistical significance: * *p* < 0.05 and ** *p* < 0.005 versus control cells. Adapted from Ref. [36].

An early example is the *i-Rheo-optical assay*, which combines a simple compression experiment with optical microscopy to determine the broadband viscoelastic properties of multicellular spheroids [36]. As illustrated in Figure 9, a microscope coverslip applies a constant compressive load while time-resolved imaging tracks the resulting morphological deformation of the spheroid. Changes in its projected geometry are used to determine the corresponding stress and strain histories, which can then be analysed directly using *i-Rheo* to obtain the frequency-dependent compressional storage and loss moduli, $E^{\prime }\left(\omega \right)$ and $E^{\prime \prime }\left(\omega \right)$, without imposing a predefined viscoelastic constitutive model. The resulting broadband moduli showed good agreement with those obtained by fitting the same experimental response with a Burgers model (Figure 9c).

The methodology was initially developed and validated using PANC-1 pancreatic cancer spheroids, including comparison with complementary nanoindentation measurements, and was subsequently applied to non-tumoral NIH/3T3 fibroblast spheroids [36]. The broadband analysis revealed distinct viscoelastic responses for the two cell lines, with PANC-1 spheroids exhibiting lower elastic and viscous contributions than NIH/3T3 spheroids. The differences were further quantified through the parameters $\eta_{1}$, $E_{1}$ and $\tau_{2}=\eta_{2}/E_{2}$, obtained by fitting the corresponding Burgers model to both the conventional model-based and *i-Rheo*-derived responses (Figure 9d–f). This application is particularly illustrative of the portability of the *i-Rheo* Framework: the primary experimental observable is an optically measured morphological evolution, rather than the output of a conventional rheometer, yet it can ultimately be converted into broadband viscoelastic information capable of resolving mechanical differences between biological systems.

A second emerging direction concerns broadband oscillatory rheometry. Optimally windowed chirp (OWCh) protocols replace sequential discrete-frequency measurements with a windowed frequency sweep, enabling a broad frequency interval to be probed within a single, short experiment [37,38]. As also discussed in the recent British Society of Rheology review [9], *i-Rheo* and OWCh offer complementary approaches to broadband linear viscoelastic characterisation.

Combining such targeted excitation with the analytical treatment of densely sampled stress and strain histories offered by *i-RheoFT* could therefore provide high-quality, well-resolved frequency-domain spectra as input to *i-Rheo-Tempo*. This integration offers a promising route towards more accurate and stable reconstruction of the time-domain relaxation modulus, although its quantitative advantages remain to be established. The integration of OWCh excitation with the *i-Rheo* analytical methodology is currently under investigation and will be reported separately.

**Figure 10:**
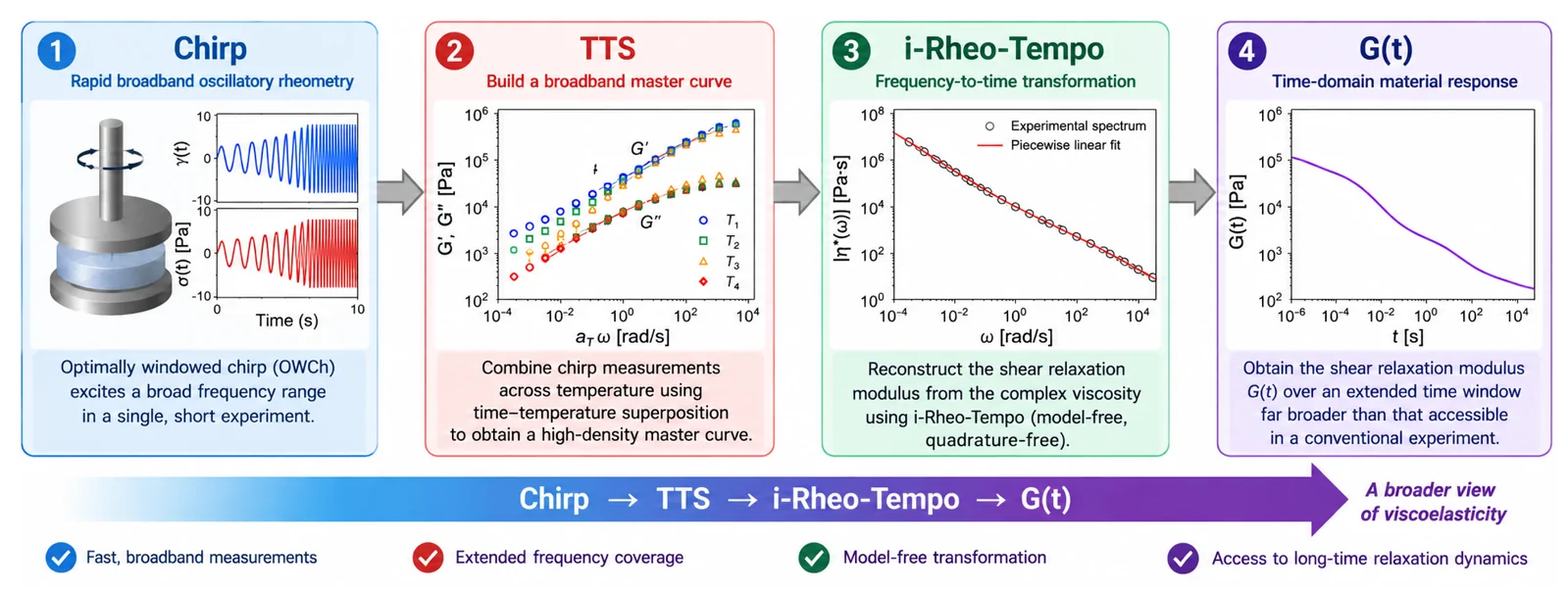
Prospective Chirp–TTS–*i-Rheo-Tempo* workflow. Schematic integration of rapid broadband chirp rheometry and time–temperature superposition (TTS) with *i-Rheo-Tempo* to reconstruct the shear relaxation modulus, G(t), over an extended time window.

A complementary opportunity arises from recent developments combining chirp rheometry with time–temperature superposition (TTS). In particular, the Thermally Optimized Ramped Chirp (TORCh) protocol combines repeated OWCh measurements with a continuous temperature ramp to generate high-density rheological master curves substantially faster than conventional discrete frequency-sweep TTS protocols [38]. Such broadband frequency-domain master curves provide a natural input for *i-Rheo-Tempo*, which can reconstruct the corresponding shear relaxation modulus directly from the complex viscosity without numerical quadrature or a predefined relaxation spectrum [8]. This prospective integration is illustrated schematically in Figure 10, in which rapid broadband oscillatory measurements are first assembled into an extended frequency-domain master curve and subsequently transformed into the time domain. In principle, this combination could provide access to relaxation dynamics over a time window far broader than that directly accessible through a conventional stress-relaxation experiment. Although this integration remains to be systematically investigated, it illustrates how complementary developments in broadband rheometry and analytical transformation could be combined to extend the experimentally accessible window of linear viscoelasticity.

Diffusing-wave spectroscopy (DWS) represents another particularly promising direction. By combining two-cell and echo measurements, modern DWS can access correlation times from approximately 10 ns to tens of seconds or longer, providing tracer dynamics over nearly nine decades in time and extending microrheological measurements to frequencies well beyond those routinely accessible by conventional mechanical rheometry [39]. Importantly, DWS-derived mean-square displacements can be converted directly into $G^{\prime }\left(\omega \right)$ and $G^{\prime \prime }\left(\omega \right)$ using the *i-Rheo* transformation, while, as demonstrated above, *i-Rheo-Tempo* provides the complementary route from the resulting broadband frequency-domain spectra back to G(t). This combination therefore opens the prospect of a bidirectional analytical framework for DWS microrheology,

$$\mathrm{MSD}\left(t\right)\;\overset{\;i\text{-}\mathrm{Rheo}\;}{\to }\;G^{*}\left(\omega \right)\;\overset{\;i\text{-}\mathrm{Rheo}\text{-}\mathrm{Tempo}\;}{\to }\;G\left(t\right),$$

in which the rheological time–frequency transformations themselves can be performed without imposing a predefined viscoelastic constitutive model.

Perhaps the most ambitious opportunity is not to extend the accessible range of any single technique but to combine complementary techniques operating at different length and time scales. Optical tweezers, dynamic light scattering (DLS), DWS and bulk rheometry probe overlapping but distinct dynamical windows and sample volumes. Their frequency-domain responses can therefore be combined to construct a substantially broader representation of the same material dynamics, while *i-Rheo-Tempo* provides a route for mapping the resulting broadband spectrum back into the time domain. Such an approach could connect local microscopic fluctuations, mesoscale dynamics and macroscopic mechanical response within a common rheological representation.

This possibility points towards a broader future role for the *i-Rheo* Framework: not simply as a family of algorithms for transforming individual experiments but as an analytical bridge between complementary measurements performed across different length, time and frequency scales. In this perspective, the ultimate objective is not merely to extend the number of decades accessible to rheological characterisation but to determine whether apparently distinct dynamical processes observed by different techniques can be assembled into a consistent description of material relaxation across scales.

A further potential application concerns lipid membranes and interfacial systems. For supported lipid bilayers, transient AFM indentation could in principle be analysed within the same time-to-frequency framework, provided that the force–indentation relation appropriately accounts for the small membrane thickness, probe geometry and mechanical influence of the underlying substrate. Similarly, transient perturbations of Langmuir monolayers could potentially provide broadband interfacial rheological information from measured area and directional surface-stress histories. However, separating dilatational and shear responses requires knowledge of the two-dimensional deformation field and the corresponding interfacial stress components. An isotropic area change primarily probes the dilatational response, whereas an approximately area-preserving in-plane deformation is required to isolate the shear response.

This distinction also requires consideration of trough geometry, barrier-induced flow, interfacial homogeneity and coupling to the underlying subphase. Indeed, the potential coupling of interfacial deformation modes is discussed in the recent British Society of Rheology review [9]. Consequently, appropriate interfacial stress–deformation relations are needed to recover surface moduli; the bulk-rheology equations cannot simply be transferred directly. In both cases, interpretation within the present linear-viscoelastic framework requires sufficiently small perturbations and an approximately stationary material state. Processes involving pore formation, membrane fusion, irreversible structural rearrangements or other evolving states would generally fall outside this linear time-invariant description, although the mechanical response preceding such events could potentially be analysed where linearity is independently established.

## 5. Conclusions

Linear viscoelasticity provides a direct window into the structure and dynamics of soft materials across a hierarchy of length and time scales. As the mechanical response of a material reflects the underlying inter- and intra-molecular interactions governing its dynamics, access to broadband viscoelastic information remains central to modern rheology, microrheology, biomechanics, and soft-matter physics. Yet, the reliable extraction of such information continues to be constrained by finite measurement windows, discrete sampling, and the limitations of conventional analytical approaches.

This Review/Perspective has traced the evolution of the *i-Rheo* Framework from its origins as a model-free transformation of creep-compliance measurements into dynamic moduli to a broader analytical methodology applicable across bulk rheology, microrheology, optical tweezers, AFM- and indentation-based characterisation, rheo-optical assays, molecular simulations, and biological systems. Despite the diversity of these experimental and computational platforms, all are connected by the same fundamental principle: the direct transformation of experimentally accessible observables into material functions without imposing predefined viscoelastic constitutive models.

The recent development of *i-Rheo-Tempo* completes this framework by establishing a reversible link between the time and frequency domains, effectively closing the time–frequency loop. Together, these developments transform what is often viewed as a numerical inversion problem into a unified analytical treatment of discretely sampled data.

Looking forward, the greatest opportunity may lie not in extending the capabilities of any individual technique but in integrating complementary measurements across length and time scales. By providing a common analytical language for broadband viscoelastic characterisation, the *i-Rheo* Framework offers a route towards connecting molecular, microscopic, and macroscopic dynamics within a single coherent description of material behaviour.

## Data Availability

No new data were created or analysed in this study. Data sharing is not applicable to this article.
